# Stabilization of Dry Sucrose Glasses by Four LEA_4 Proteins from *Arabidopsis thaliana*

**DOI:** 10.3390/biom11050615

**Published:** 2021-04-21

**Authors:** Dirk K. Hincha, Ellen Zuther, Antoaneta V. Popova

**Affiliations:** 1 Max-Planck-Institut für Molekulare Pflanzenphysiologie, Am Mühlenberg 1, 14476 Potsdam, Germany; hincha@mpimp-golm.mpg.de; 2 Institute of Biophysics and Biomedical Engineering, Bulgarian Academy of Sciences, 1113 Sofia, Bulgaria

**Keywords:** FTIR spectroscopy, sucrose, dehydration, LEA proteins, sugar glass, glass transition, hydrogen bonding

## Abstract

Cells of many organisms and organs can withstand an (almost) total water loss (anhydrobiosis). Sugars play an essential role in desiccation tolerance due to their glass formation ability during dehydration. In addition, intrinsically disordered LEA proteins contribute to cellular survival under such conditions. One possible mechanism of LEA protein function is the stabilization of sugar glasses. However, little is known about the underlying mechanisms. Here we used FTIR spectroscopy to investigate sucrose (Suc) glass stability dried from water or from two buffer components in the presence of four recombinant LEA and globular reference proteins. Buffer ions influenced the strength of the Suc glass in the order Suc < Suc/Tris < Suc/NaP. LEA proteins strengthened the sugar H-bonded network and the molecular structure in the glassy state. The position of νOH peak and the wavenumber–temperature coefficient (WTC_g_) provided similar information about the H-bonded network. Protein aggregation of LEA proteins was reduced in the desiccation-induced Suc glassy state. Detailed knowledge about the role of LEA proteins in the stabilization of dry sugar glasses yields information about their role in anhydrobiosis. This may open the possibility to use such proteins in biotechnical applications requiring dry storage of biologicals such as proteins, cells or tissues.

## 1. Introduction

Sugars, in particular disaccharides such as sucrose (Suc) and trehalose, play an important role in the desiccation tolerance of most, but not all [1,2] anhydrobiotic organisms (see [3] for a review). In the anhydrous state, two major mechanisms exist by which sugars can stabilize cells or cellular components. On the one hand, sugars can effectively protect proteins and membranes from damage by H-bonding with amino acid side chains in proteins or hydrophilic head groups of lipids, thereby replacing water molecules that were lost from the hydration sphere during drying (see for example, [4,5,6,7]). On the other hand, the protective properties of sugars during desiccation are based on their ability to vitrify during drying instead of crystallizing. Thereby they provide a solid matrix within cells that prevents cell collapse and diffusion-driven damage to cellular constituents [8,9,10].

Sugar glasses show a temperature-dependent transition from the glassy state (i.e., an amorphous quasi-solid state with very high viscosity) to a rubbery melted state, with a well-defined glass transition temperature (T_g_). For dry Suc, T_g_ values of 70 °C [11], 66.5 °C [12], 65 °C [13], 60 °C [14], 57 °C [15], and 56.6 °C [16] have been reported. These differences can most likely be attributed to different methods used for the determination of T_g_ and to minor differences in sample water content, as water is an effective plasticizer of sugar glasses [17,18].

The properties of sugar glasses can also be modulated by the presence of various inorganic ions, such as phosphate [19,20,21], via strengthening the H-bonded network among sugar molecules and thereby increasing the T_g_ of the mixtures. In addition, there is evidence that intrinsically disordered proteins (IDPs) may influence glass transition behavior and survival of anhydrobiotic organisms, such as tardigrades [22], or of specific anhydrobiotic life stages of plants, such as pollen and seeds [9,23]. In many organisms, intrinsically disordered late embryogenesis abundant (LEA) proteins have been associated with desiccation tolerance [24,25]. In vitro studies have shown that some LEA proteins participate in the formation of H-bonded networks with sugars, thus increasing the stability of the sugar glasses formed during drying, as indicated by an increased T_g_ [14,26,27]. This increase in the stability of sugar glasses could improve the preservation of cells in the dry state, particularly at elevated temperatures.

LEA proteins represent a large and heterogeneous group of proteins that have first been described in plant seeds during the late stages of seed development when seeds become desiccation-tolerant [28]. They are, however, not seed-specific but have also been found in vegetative plant tissues under various environmental stress conditions and in some desiccation-tolerant invertebrate animals and microorganisms [24,25].

A total of 51 genes encoding LEA proteins from nine different Pfam families have been previously identified in *Arabidopsis thaliana* [29], with the LEA_4 family being the largest and most heterogeneous. However, LEA_4 proteins have diverse properties, as, e.g., tissue and subcellular localization. They share common features, such as low sequence complexity, high hydrophilicity and a lack of secondary structure under fully hydrated conditions [30]. Interestingly, all LEA_4 proteins investigated to date fold into the α-helical structure during dehydration [30,31,32]. Functionally, LEA_4 proteins have been associated with stabilizing enzymes [33,34,35,36] and membranes [30,31,36,37,38,39] during freezing and drying. A role of two LEA_4 proteins from the plant *Typha latifolia* and the invertebrate animal *Polypedilum vanderplanki* in sugar glass stabilization has also been reported [14,27]. However, to the best of our knowledge, no information about the effect of the more extensively studied *Arabidopsis* LEA_4 proteins on sugar glasses has been published.

Here we present a systematic FTIR spectroscopy study reporting on the ability of the four LEA_4 proteins LEA11, COR15A (LEA24), LEA25 and LEA29 from *Arabidopsis thaliana* [29] to stabilize Suc glasses in the dry state. For comparison, we included two globular proteins, bovine serum albumin and β-lactoglobulin (BSA and LG). In addition, we investigated the effects of two commonly used buffer substances (phosphate and Tris) on Suc glass properties, compared to samples dried from solutions that contained only Suc and water. The OH peak in the spectral region 3600–3000 cm^−1^ was used to determine T_g_ and the stability of the Suc glass as affected by the presence of proteins. The position of νOH peak and the wavenumber–temperature coefficient in the glassy state (WTC_g_) of pure Suc and Suc/protein mixtures were compared to evaluate the strength of H-bonding interactions and molecular packing in the Suc glass in the dry state. The fingerprint region of Suc (1500–900 cm^−1^) was analyzed to detect LEA protein-induced alterations in the molecular structure of Suc. By comparing the amide I peak of dry recombinant LEA proteins and Suc/LEA protein mixture, changes in protein secondary structure and aggregation of LEA proteins were measured.

## 2. Materials and Methods

### 2.1. Materials

Sucrose (Suc), Tris (tris(hydroxymethyl)aminomethane), Na_2_HPO_4_, NaH_2_PO_4_, β-lactoglobulin (LG) and fatty acid-free bovine serum albumin (BSA) were obtained from Sigma (Taufkirchen, Germany).

### 2.2. LEA Proteins

The genes encoding LEA11 (AT2G03740), COR15A (LEA24; AT2G42450), LEA25 (AT2G42560) and LEA29 (AT3G15670) from *Arabidopsis thaliana* were expressed in the *E. coli* strains Rosetta or BL21 DE3. The corresponding cDNA clones were obtained from the RIKEN (Tokyo, Japan) RAFL collection [40,41] and were cloned without the sequences encoding N-terminal signal peptides into expression vectors yielding untagged protein (LEA11) or His-tagged proteins. Protein purification and TEV protease-mediated removal of His-tags were performed as described previously [30,31,42]. Protein purity was evaluated by SDS–PAGE [43] and Coomassie blue staining. Purified proteins were lyophilized and stored at −20 °C until use. Protein concentration was determined photometrically using the sequence-specific extinction coefficient at 280 nm calculated with the ProtParam tool on the ExPASy server [30].

### 2.3. Sample Preparation

Suc was dissolved at a concentration of 10 mg/mL in either H_2_O or 10 mM Tris or sodium phosphate (NaP) buffer at pH 7.4. Proteins were dissolved separately in the respective solutions at defined concentrations. Afterward, Suc and protein samples were mixed to obtain Suc/protein mass ratios of 5:1 or 2:1. Pure proteins without Suc dissolved in H_2_O, Tris or NaP buffer were measured at a concentration of 2 mg/mL.

### 2.4. FTIR Spectroscopy

Spectra of all samples were recorded with a PerkinElmer GX2000 FTIR spectrometer (Rodgau, Germany). Samples were prepared by applying 50 μL of the different test solutions to CaF_2_ windows and afterward drying them over silica gel at 28 °C for 24 h. Dry samples were placed in a vacuum sample holder equipped with temperature control (Specac Eurotherm, Worthington, UK) and placed in the infrared beam as described in detail previously [44,45]. Samples were kept under vacuum at 100 °C for 30 min to remove residual water entrapped in the Suc glass, thus allowing the investigation of essentially anhydrous samples [4,45]. The temperature was rapidly decreased to −30 °C using liquid nitrogen and subsequently slowly (1 °C/min) increased to 140 °C (well below the melting point of crystalline Suc at 186 °C). Sample temperature was monitored using a thermocouple attached to the sample window. Every minute, two spectra were recorded and co-added in the spectral region of 4000–900 cm^−1^ with a nominal resolution of 4 cm^−1^. Spectra were analyzed using the PerkinElmer Spectrum 5.0.1 software.

The position of the OH-stretching vibration peak (νOH) was determined as the midpoint of the peak at 80% of its maximal height between 3600 cm^−1^ and 3000 cm^−1^ after normalization of the peak height to unity. Melting curves of Suc glasses of different compositions were constructed by plotting the position of νOH versus the temperature. The melting temperature of Suc glasses (T_g_) was determined as the intersection of the linear regressions fitted to the glassy (8–50 °C) and liquid (100–120 °C) states [14,15,20].

Additionally, the position of νOH at 30 °C, corresponding to the glassy state, was determined to evaluate geometric parameters of H-bonding interactions, as it was previously shown to correlate with O–H bond length and O…O distance, informative about the strength of the H-bonded network [46]. To evaluate the molecular packing density of the Suc glasses and the strength of H-bonding interactions between Suc molecules, the wavenumber–temperature coefficient in the glassy state (WTC_g_) was calculated as the slope of the linear regression fitted to the glassy state [20].

The fingerprint region of FTIR spectra (1500–900 cm^−1^), originating from symmetrical deformation vibrations of CH_2_ groups, as well as from C–O-stretching vibrations of sugars [47], was analyzed to evaluate differences in the molecular geometry of Suc caused by the presence of buffer components and/or proteins. In addition, the region between 1500 cm^−1^ and 1200 cm^−1^ contains several vibration peaks attributed mainly to CH_2_ vibration modes [48]. The vibration peak at around 1460 cm^−1^ is assigned to scissoring modes of CH_2_, and the peaks at 1343 cm^−1^ and 1278 cm^−1^ to rocking vibration modes of CH_2_ [48], while the vibration peak at 1209 cm^−1^ is indicative of the OH deformation asymmetric ring mode [49]. The peaks in the spectral region of 1200–900 cm^−1^ originate predominantly from combinations of CO (υCO)-stretching and OH-bending (δCOH) vibrations [20,47].

Protein secondary structure was evaluated by comparing amide I peaks (1700–1600 cm^−1^) in the absence and presence of Suc [50]. Spectra were recorded at 30 °C by co-adding 64 spectra. The broad amide I peak originates from vibrations from the backbone of the protein molecule and is composed of several overlapping component vibration peaks attributed to α-helices (around 1660–1650 cm^−1^), β-sheets (1640–1620 cm^−1^), turns (1670–1660 cm^−1^), and unstructured elements (1650–1640 cm^−1^) [51,52,53]. The formation of β-sheet aggregates is characterized by a peak at about 1620 cm^−1^ [54,55].

## 3. Results

### 3.1. FTIR Spectra of Dry Suc

**Figure 1:**
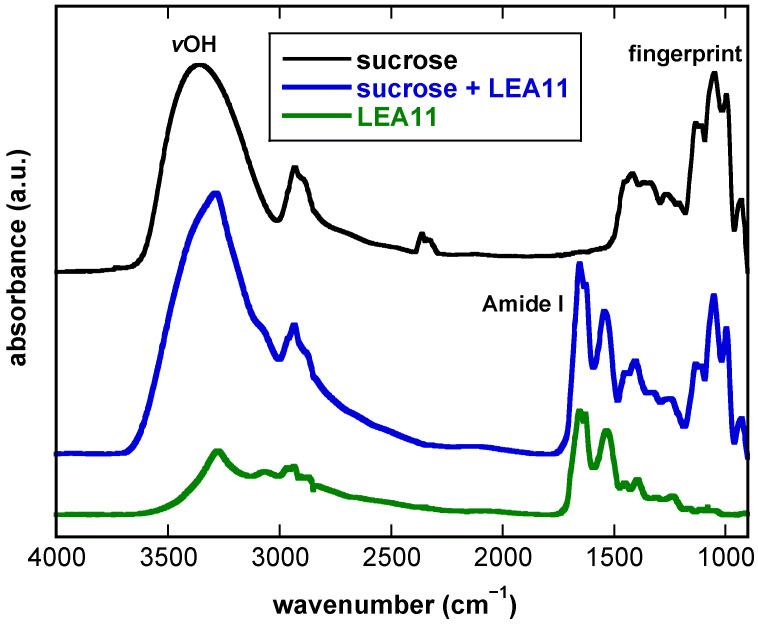
FTIR absorbance spectra of dry samples prepared from 10 mg/mL Suc, from 10 mg/mL Suc and 5 mg/mL LEA11, and from 2 mg/mL LEA11. Suc and LEA11 were dissolved in water, and samples were then dried on CaF_2_ windows. Spectra were recorded at 30 °C. The OH-stretching vibration peak (νOH) of the sugar, the amide I peak of the protein and the fingerprint region of the sugar spectrum are indicated. a.u.—arbitrary units.

In the FTIR spectrum of pure Suc (Figure 1), two main absorbance regions were observed: the broad OH vibration peak (νOH) in the spectral region between 3600 cm^−1^ and 3000 cm^−1^ and the fingerprint region (1500–900 cm^−1^). The fingerprint region in the FTIR spectra of sugars is due to several different vibration modes originating from the sugar backbone, and its contour is a characteristic feature of different sugar molecules. In addition, the peak observed at around 2900 cm^−1^ originates from several symmetric and asymmetric stretching vibrations of sugar CH_2_ groups [48]. The lack of OH-scissoring vibrations at around 1645 cm^−1^ provides evidence that under our experimental conditions, our samples were completely anhydrous.

The broad νOH peak originates from H-bonding interactions between different OH groups of Suc, with different lengths and orientations. It is shifted to lower wavenumbers with an increase in the number and strength of these interactions. The νOH peak of pure Suc at 30 °C (3346 cm^−1^) was shifted to lower wavenumbers (3314 cm^−1^) in the presence of LEA11, indicating the formation of additional H-bonds between sugar and protein. In addition, the νOH peak of the Suc/LEA11 mixture was narrower than the peak observed in pure Suc, indicating a more uniform length and orientation of H-bonds. In the spectrum of the pure, dry protein, the amide I and amide II peaks, originating from vibrations of the amide bonds in the protein backbone [52,56], are prominent (Figure 1). The amide peaks were also clearly resolved in the spectra of the Suc/LEA11 mixture, which made it possible to analyze the peaks of both sugar and protein simultaneously. In the spectra of LEA11, νOH is also observed, assigned to the intrinsic OH groups of proteins, but it is weak compared to the massive OH peak of Suc due to a much higher relative number of OH groups [14].

### 3.2. The Presence of Buffer Components Influences the Properties of Dry Suc Glasses

**Figure 2:**
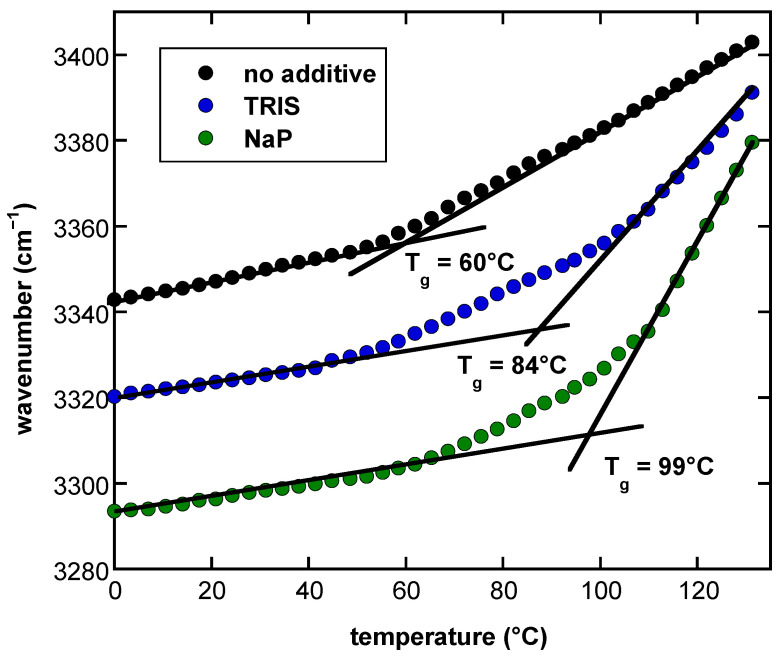
Melting curves of dry Suc glasses determined from the temperature-induced shift of the position of the νOH peak in FTIR spectra. Suc at a final concentration of 10 mg/mL was dissolved in pure H_2_O, in Tris or NaP buffer (10 mM, pH 7.4) and dried on CaF_2_ windows. Glass transition temperatures (T_g_) were determined from the intersection of fitted regression lines in the glassy state at low and the melted state at higher temperatures. T_g_ of the different samples is indicated.

T_g_ of Suc glasses was determined from melting curves constructed by plotting the position of the νOH peak against temperature (Figure 2). Suc was either dried from pure water or from 10 mM NaP or Tris buffer. At 0 °C, the position of the νOH peak was located at 3342 cm^−1^ when the sugar was dried from pure water. The presence of Tris shifted the νOH position to 3320 cm^−1^, while it was shifted to 3293 cm^−1^ in the presence of NaP. These shifts indicate increased H-bonding interactions in the Suc glasses in the presence of buffer components, with a stronger effect from NaP than from Tris.

With increasing temperature, the position of the νOH peak was shifted to higher wavenumbers due to the decreasing strength of H-bonding interactions (Figure 2). Two distinct linear regions with different slopes were observed for the melting curve of pure Suc that are characteristic of the glassy and the melted state, respectively. The intercept of these linear phases defines T_g_ [14,15,20]. In the melting curves of Suc dried from buffer solutions, the transfer from the glassy to the melted state was more gradual. Analysis of the intercepts of the linear regressions of the glassy and melted states of the melting curves indicated that the T_g_ of pure dry Suc was 60 °C, in agreement with earlier data obtained using the same method [14]. The presence of Tris in the Suc glass increased T_g_ to 84 °C, while the presence of NaP resulted in a T_g_ of 99 °C.

### 3.3. Addition of Proteins Influences the Position of the νOH Peak of Suc in the Glassy State

Here we present a comparative study on the ability of four LEA proteins from *Arabidopsis thaliana* to participate in forming a common H-bonded network with Suc and to stabilize the Suc glass in a fully dehydrated state. The three closely related LEA proteins LEA11, COR15A and LEA25 form a small subgroup of the LEA_4 family, share a conserved central sequence domain and are largely unstructured in a fully hydrated state. Structural analysis of these LEA proteins indicated folding into the more ordered secondary structure, mainly α-helical elements, in the presence of osmolytes, such as Suc, glycerol and ethylene glycol, in organic solvents as 2,2,2-trifluoroethanol (TFE) and especially under conditions of severe water loss [30,36]. LEA29 also belongs to the LEA_4 family but is not closely related to LEA11, COR15A and LEA25.

**Figure 3:**
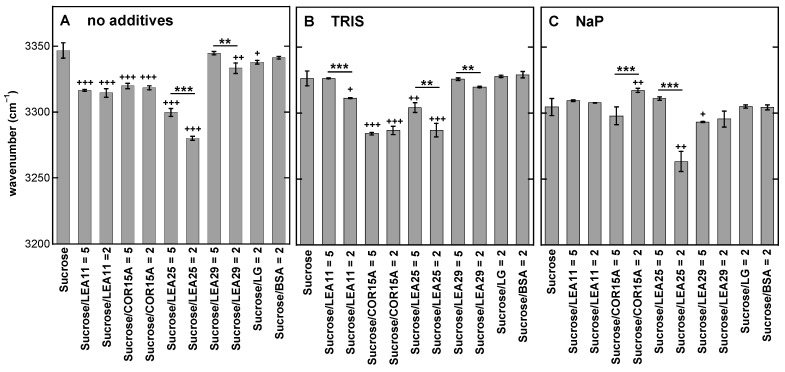
Position of the νOH peak of dry Suc in the glassy state at 30 °C. Samples contained either pure Suc or, in addition, the proteins LEA11, COR15A, LEA25, LEA29, β-lactoglobulin (LG) or bovine serum albumin (BSA). All solutions contained 10 mg/mL Suc and were prepared in H_2_O (**A**), 10 mM Tris (**B**) or 10 mM NaP buffer (pH 7.4) (**C**). In addition, samples contained proteins at the indicated Suc/protein mass ratios. Samples were dried on CaF_2_ windows. The bars indicate the means from at least three samples ± standard deviation. Statistically significant differences between the values determined at the two different Suc/LEA mass ratios are indicated by *, while significant differences between the values obtained in the presence of the different proteins and pure Suc are indicated by + ( + <0.05; **, ++ ˂0.01, ***, +++ ˂0.001).

As the data presented in Figure 1 and Figure 2 clearly indicated an influence of both the buffer component and the LEA protein on the H-bonded networks in Suc glasses, we investigated these effects in a full factorial design. It combined no buffer or Tris and NaP solutions with four different LEA proteins at two different Suc/protein ratios and two globular reference proteins, resulting in a total of 33 different sample compositions (Figure 3). As an example for determining the wavenumber of the νOH peak, the melting curves of Suc/LEA11 dehydrated from H_2_O, Tris or NaP are shown in Supplemental Figure S1.

The νOH peak of pure Suc at 30 °C was located at 3346 cm^−1,^ and the addition of Tris or NaP resulted in an overall shift of the νOH peaks of all samples to lower wavenumbers compared to samples without buffer. In addition, all four LEA proteins induced νOH peak shifts of various magnitudes in the same direction, while the globular reference proteins had only negligible effects. However, the shifts in νOH were generally stronger and more often significant in the absence of buffer components than in the presence of Tris. In the presence of NaP, only LEA25 at a low Suc/protein ratio resulted in a strong downshift of the νOH peak. Interestingly, in the presence of NaP, we even observed a significant increase in νOH at a low Suc/COR15A ratio that may indicate a disruption of H-bonding between Suc and NaP by this protein in the Suc glass.

### 3.4. Strength of H-Bonding Interactions in Dry Suc Glasses

Another important parameter that characterizes the stability of sugar glasses is WTC_g_, which is a further indicator of the strength of the H-bonded network in the glass [20], as it shows how strongly νOH is influenced by temperature. If an additive (buffer ions or protein) lowers WTC_g_, this indicates stronger H-bonding because the H-bonds are less influenced by temperature.

**Figure 4:**
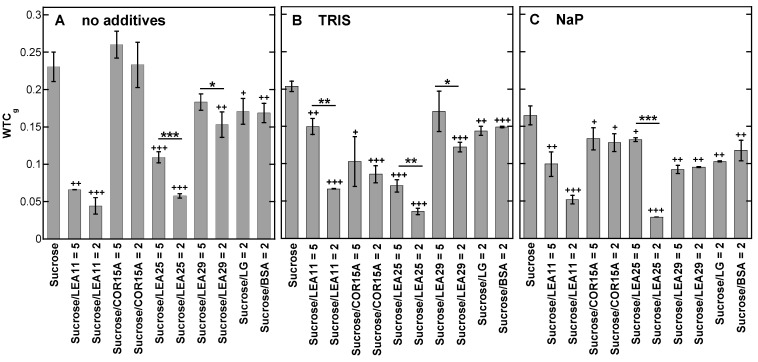
Wavenumber-temperature coefficients in the glassy state (WTC_g_) of dry Suc. Samples contained either pure Suc or, in addition, the proteins LEA11, COR15A, LEA25, LEA29, β-lactoglobulin (LG) or bovine serum albumin (BSA). All solutions contained 10 mg/mL Suc and were prepared in H_2_O (**A**), 10 mM Tris (**B**) or 10 mM NaP buffer (pH 7.4) (**C**). In addition, samples contained proteins at the indicated Suc/protein mass ratios. Samples were dried on CaF_2_ windows. The bars indicate the means from at least three samples ± standard deviation. Statistically significant differences between the values determined at the two different Suc/LEA mass ratios are indicated by *, while significant differences between the values obtained in the presence of the different proteins and pure Suc are indicated by + (*, + ˂0.05; **, ++ ˂0.01, ***, +++ ˂0.001).

In the absence of proteins, WTC_g_ was highest when Suc was dried from H_2_O, and it decreased in the presence of Tris and NaP from 0.230 to 0.203 and 0.164, respectively (Figure 4), indicating that both buffer components increased the strength of the H-bonds in the Suc glasses. The addition of proteins (both LEAs and globular proteins) generally induced a reduction in WTC_g_, indicating that the H-bonding interactions between Suc and proteins were stronger than between Suc OH groups and/or Suc and buffers. Unlike the influence on the position of the νOH peak (Figure 3), the effects of the proteins on WTC_g_ were still pronounced in the presence of NaP, indicating that these two measures provide different information on the stability of the Suc glasses. This is emphasized because the position of the νOH peak was hardly influenced by the globular proteins, while they had a significant effect on WTC_g_ in the absence and presence of buffer components. In most cases, we observed a concentration dependence of the effects of the LEA proteins, although the differences between the two concentrations were not always significant. In Suc glasses dried from all solutions (no additives, Tris, NaP), LEA11 and LEA25 induced consistently strong reductions in WTC_g_, while LEA29 had smaller effects. Interestingly, COR15A showed no effect in the absence of buffer components, a small effect in the presence of NaP and the strongest reduction in WTC_g_ in the presence of Tris.

### 3.5. Effect of Buffer Components and Proteins on T_g_

**Figure 5:**
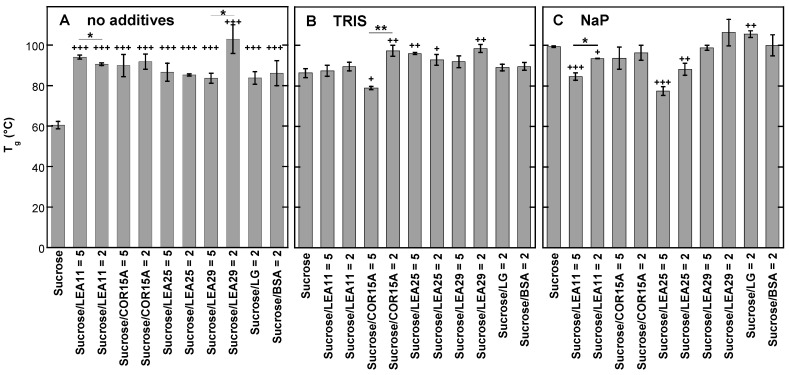
Glass transition temperature (T_g_) of dry Suc. Samples contained either pure Suc or, in addition, the proteins LEA11, COR15A, LEA25, LEA29, β-lactoglobulin (LG) or bovine serum albumin (BSA). All solutions contained 10 mg/mL Suc and were prepared in H_2_O (**A**), 10 mM Tris (**B**) or 10 mM NaP buffer (pH 7.4) (**C**). In addition, samples contained proteins at the indicated Suc/protein mass ratios. Samples were dried on CaF_2_ windows. The bars indicate the means from at least three samples ± standard deviation. Statistically significant differences between the values determined at the two different Suc/LEA mass ratios are indicated by *, while significant differences between the values obtained in the presence of the different proteins and pure Suc are indicated by + (*, + ˂0.05; **, ++ ˂0.01, +++ ˂0.001).

As already indicated in Figure 2, the presence of Tris or NaP strongly increased T_g_ of dry Suc compared to the sugar without any additives. The presence of all LEA proteins and the two globular proteins increased T_g_ in Suc glasses without buffer components to the same extent as for Suc alone in the presence of Tris (Figure 5). When Tris was present in the glasses, the addition of COR15A, LEA25 and LEA29 led to a further increase in T_g_, to levels similar to T_g_ in the presence of NaP without protein, while LEA11 and the globular proteins had no effect. In the presence of NaP, which increased T_g_ by 39 °C (Figure 2), the addition of LEA11 and LEA25 resulted in an unexpected decrease in T_g_, while the other proteins only had minor effects.

**Figure 6:**
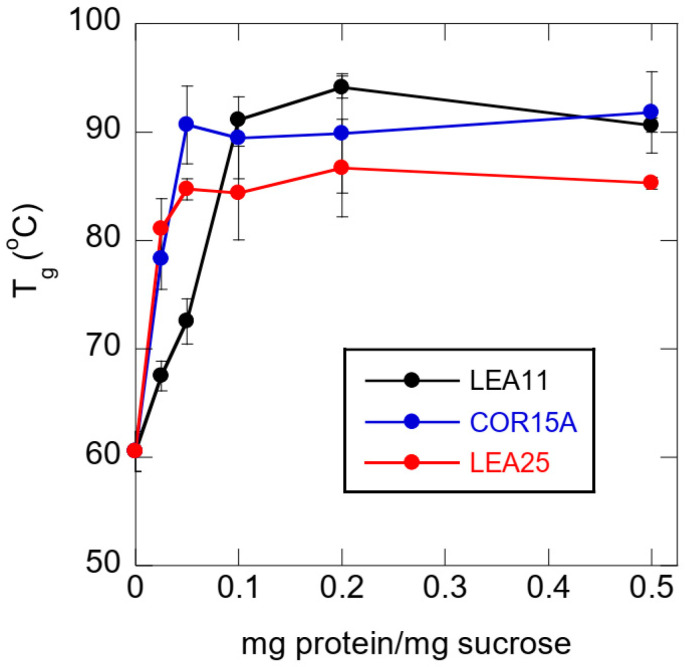
Dependence of the glass transition temperature (T_g_) of dry Suc on the ratio mg protein/mg Suc. Before dehydration, samples contained 10 mg/mL Suc in H_2_O and, in addition, the respective amount of protein to reach the indicated ratios, mg protein/mg Suc. Samples were dried on CaF_2_ windows. Values are means from at least three samples ± standard deviation.

The stabilizing effect of all four LEA proteins on Suc glasses was strongest in the absence of any buffer ions (Figure 5). To quantify these effects in more detail, we dried Suc from H_2_O with the addition of increasing concentrations of the three closely related proteins LEA11, COR15A and LEA25 and determined T_g_ (Figure 6). All three LEA proteins increased T_g_ already at the lowest concentration (0.25 mg/mL) and at a concentration of 0.5 mg/mL (ratio Suc/LEA 20:1). The effects of COR15A and LEA25 on T_g_ were already maximal. LEA11 was less effective, and the maximal effect was reached at 2 mg/mL (ratio Suc/LEA 5:1). At saturating concentrations, T_g_ reached between 90 °C and 95 °C with LEA11 and COR15A, while for LEA25, the maximal T_g_ was around 85 °C.

**Figure 7:**
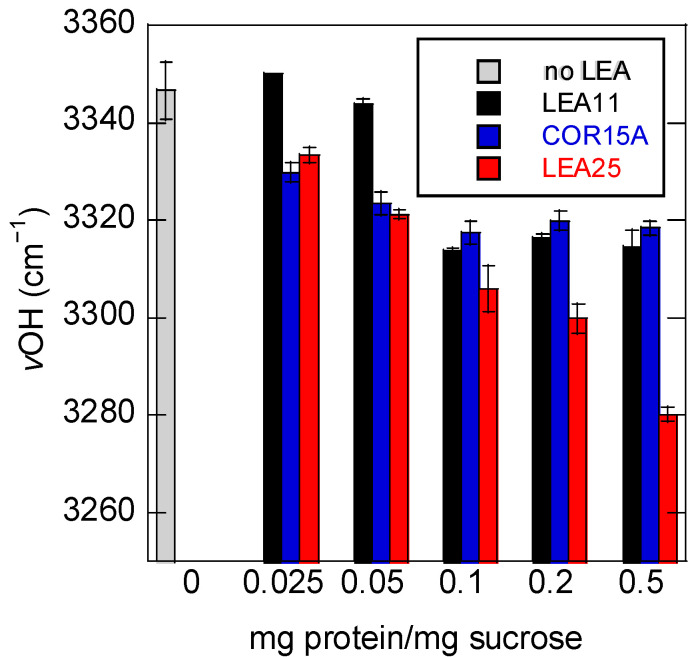
Dependence of the νOH peak position of dry Suc glass on the ratio mg protein/mg Suc. Before dehydration, samples contained 10 mg/mL Suc in H_2_O and, in addition, the respective amount of protein to reach the indicated ratios, mg protein/mg Suc. Samples were dried on CaF_2_ windows, and spectra were recorded at 30 °C. Values are means from at least three samples ± standard deviation.

To evaluate the relationship between LEA protein concentration and νOH, we determined the νOH peak position at 30 °C in all samples (Figure 7). Interestingly, the effect of LEA11 on νOH was smaller at low concentrations than the other two proteins, similar to the behavior of T_g_. However, while at higher concentrations, νOH remained at about 3320 cm^−1^ in the presence of LEA11 and COR15A, the presence of LEA25 reduced νOH to 3280 cm^−1^, although T_g_ was very similar in the presence of COR15A and LEA25. This indicates that H-bonding patterns and T_g_ are not closely linked in Suc glasses in the presence of different LEA proteins.

**Figure 8:**
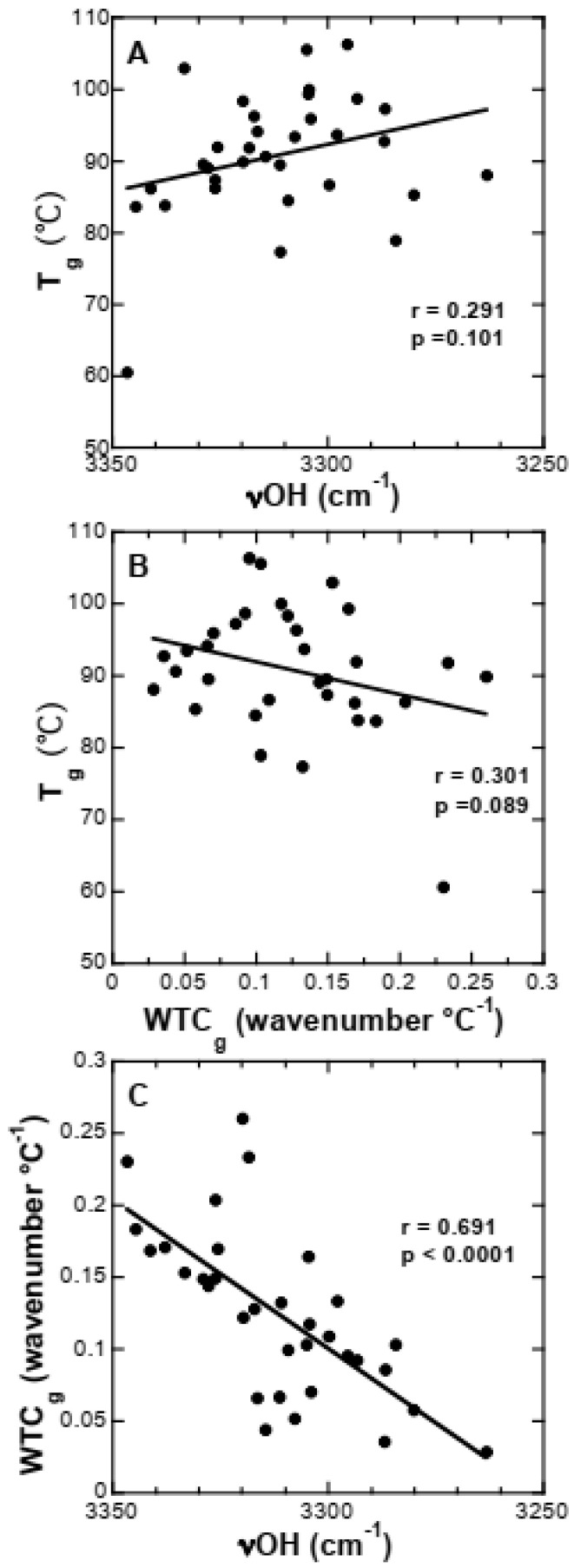
Correlation analysis between Tg, WTCg and νOH. Data from all samples analyzed in this study (Suc dissolved in pure H2O, Tris and NaP containing no proteins, LEA proteins or globular reference proteins) were used to correlate Tg with νOH (**A**), Tg with WTCg (**B**), and WTCg with νOH (**C**). The calculated r- and p-values are indicated in each panel.

Since the data shown in Figure 3, Figure 4, Figure 5, Figure 6 and Figure 7 indicated that the three parameters used to characterize the Suc glasses (νOH, WTC_g_ and T_g_) did not provide consistent information, we systematically correlated all measurements for these parameters with each other (Figure 8). We found that T_g_ was not significantly correlated with the two parameters measuring H-bonding strength (νOH and WTC_g_), although it is usually assumed that H-bonding determines T_g_. On the other hand, the measures of H-bonding strength were highly significantly correlated, indicating that either one may be used to characterize the physical properties of sugar glasses.

### 3.6. Influence of Buffer Components and Proteins on the Fingerprint Region of FTIR Spectra from Dry Suc Glasses

**Figure 9:**
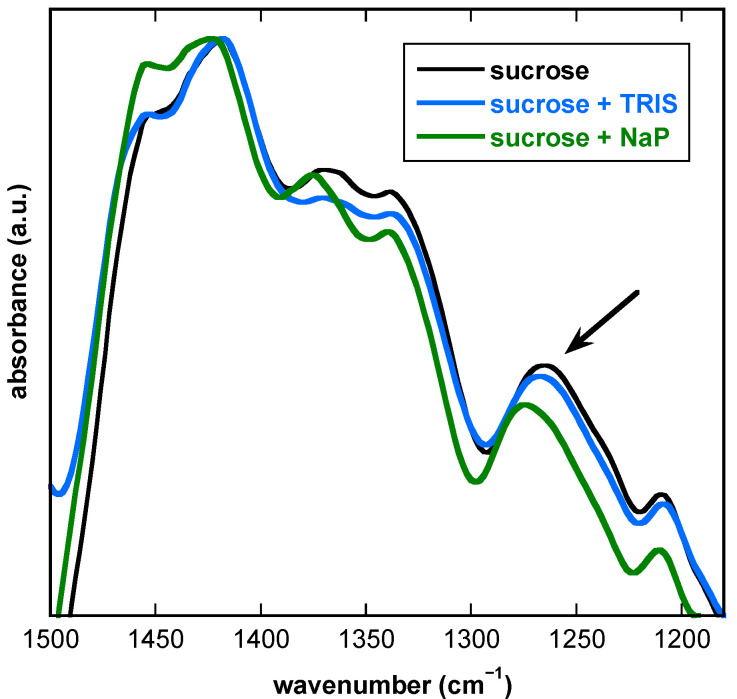
FTIR spectra of dry Suc glass in the fingerprint region (1500–1150 cm^−1^) at 30 °C. Samples contained 10 mg/mL Suc in pure H_2_O, or in 10 mM Tris or 10 mM NaP (pH 7.4). Samples were dried on CaF_2_ windows, and spectra were recorded at 30 °C. Spectra were normalized to the absorbance at 1420 cm^−1^. The vibration peak at about 1268 cm^−1^ is indicated with an arrow.

In the fingerprint region, the two peaks at 1452 cm^−1^ and ~1420 cm^−1^ (Figure 9) originate from CH_2_-scissoring vibrations of Suc molecules [48]. The presence of Tris had no effect on these peaks, but in the presence of NaP, the peak at 1418 cm^−1^ was shifted to 1424 cm^−1,^ and the relative intensity of the peak at 1452 cm^−1^ was increased. Likewise, two peaks at ~1366 cm^−1^ and 1336 cm^−1^ were resolved, originating from rocking modes of CH_2_ [48]. While both Tris and NaP did not affect the peak at 1336 cm^−1^, the peak at 1366 cm^−1^ was shifted to 1372 cm^−1^ only in the presence of NaP. Similarly, the peak located at 1268 cm^−1^ in pure Suc, also originating from rocking modes of CH_2_ [48,49], was shifted up-field by 1.5 cm^−1^ in the presence of Tris and by 8 cm^−1^ in the presence of NaP. Finally, the position of the peak at 1220 cm^−1^, originating from deformation modes of OH groups [48], was not affected by the addition of Tris or NaP to the Suc solution before drying.

**Figure 10:**
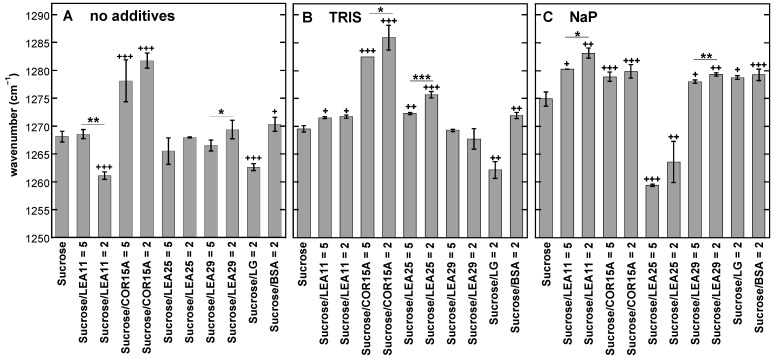
Influence of the presence of buffers and proteins on the position of the absorbance peak located at about 1268 cm^−1^ in a pure Suc glass. Samples contained either pure Suc or, in addition, the proteins LEA11, COR15A, LEA25, LEA29, β-lactoglobulin (LG) or bovine serum albumin (BSA). All solutions contained 10 mg/mL Suc and were prepared in H_2_O (**A**), 10 mM Tris (**B**) or 10 mM NaP buffer (pH 7.4) (**C**). Samples were dried on CaF_2_ windows, and spectra were recorded at 30 °C. Values are means from at least three parallel samples ± standard deviation. Statistically significant differences between the values determined at the two different Suc/LEA mass ratios are indicated by *, while significant differences between the values obtained in the presence of the different proteins and pure Suc are indicated by + (*, + ˂0.05; **, ++ ˂0.01, ***, +++ ˂0.001).

Since the peak at ~1268 cm^−1^ showed the strongest shift in response to the presence of NaP, we analyzed the effects of proteins on its position in more detail (Figure 10). COR15A induced the strongest up-shift of this peak by 13 cm^−1^ in the absence of buffer components and by 16 cm^−1^ in the presence of Tris, while the peak was only shifted by 5 cm^−1^ in the presence of NaP. LEA11, on the other hand, showed the strongest effect in the presence of NaP. Interestingly, while LEA25 did not affect the position of this peak in the absence of buffer components, it induced a moderate up-shift in the presence of Tris, but a massive downshift in the presence of NaP, altering the peak position to around 1260 cm^−1^, i.e., to a lower position than in the absence of any buffer component or protein. A similar effect was observed for the globular protein LG without buffer components or in the presence of Tris. BSA, on the other hand, induced a moderate up-shift of this peak under all three conditions.

### 3.7. The Presence of Both Buffer Components and Suc Influences the Extent of LEA Protein Aggregation in the Dry State

**Figure 11:**
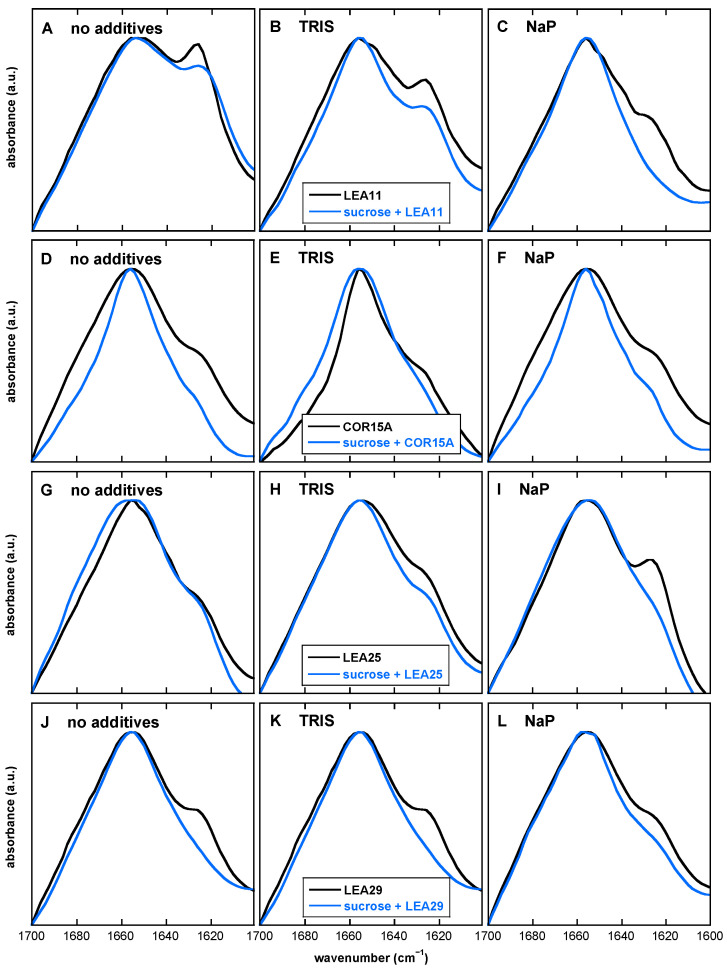
Normalized amide I peaks from FTIR spectra of dry LEA11 (**A**–**C**), COR15A (**D**–**F**), LEA25 (**G**–**I**) and LEA29 (**J**–**L**). Samples contained the LEA proteins in the absence or presence of Suc at a mass ratio 2 in H_2_O (no additive) (**A**,**D**,**G**,**J**), 10 mM Tris (**B**,**E**,**H**,**K**), or 10 mM NaP (pH 7.4) (**C**,**F**,**I**,**L**) as indicated in the panels. Samples were dried on CaF_2_ windows, and spectra were recorded at 30 °C.

Alterations in the secondary structure of proteins in the dry state can be easily followed by analyzing the amide I peak in FTIR spectra in the region between 1700 cm^−1^ and 1600 cm^−1^. Some LEA proteins tend to form aggregates in response to desiccation, giving rise to a peak at 1620 cm^−1^ [32,50,57]. The amide I peak of all four LEA proteins was located at about 1656 cm^−1^, indicating the expected folding of the disordered proteins into α-helices upon drying [30,31], irrespective of the presence of buffer ions or Suc (Figure 11).

In all cases, we observed a shoulder on the amide I peak at around 1620 cm^−1^ that indicates the desiccation-induced formation of β-sheet aggregates. The extent of aggregation, however, varied among LEA proteins and was influenced by the presence of buffer components and Suc. In the absence of any additives, LEA11 showed the strongest aggregation, followed by LEA29. In most cases, the addition of buffer components reduced aggregation, with the exception of LEA25, which showed increased aggregation in the presence of NaP. The presence of Suc suppressed aggregation in all proteins. In most cases, this suppression was complete. However, LEA11 still showed strong aggregation in the Suc glass without additive, which was further reduced or completely suppressed with the addition of Tris or NaP, respectively.

## 4. Discussion

### 4.1. Tris and Phosphate Buffer Components Increase Number and Strength of H-Bonding Interactions in Suc Glasses and Alter the Molecular Structure of Suc in the Dry State

The broad νOH peak of pure sugars in the spectral region 3600–3000 cm^−1^ originates from intermolecular H-bonding interactions between OH groups of sugars and represents different overlapping vibration peaks, corresponding to hydrogen bonds with different lengths and orientations. This peak position indicates the number and strength of H-bonds formed between OH groups of Suc in the Suc glass. With increasing temperature, the H-bonded network between Suc molecules is less densely packed, and the dry Suc glass is transformed from a glassy to a melted state. This results in a shift of the position of the νOH peak of pure Suc to higher wavenumbers. The length of the hydrogen bonds in the melted state is always longer than that in the glassy state under the same conditions, which denotes a decrease in hydrogen bonding strength with increased temperature [15].

A higher number of H-bonds was formed between Suc and phosphate ions during full dehydration of Suc/NaP, and the strength of these bonds was much stronger than the intermolecular ones between the OH groups of Suc, resulting in a 39 °C higher T_g_ of Suc/NaP than that of the pure Suc glass. These results are in good agreement with published data obtained using FTIR spectroscopy, which indicated that intermolecular sugar–sugar H-bonds had been disrupted in the presence of phosphate, and new ones with higher strength and with longer lifetime had been preferentially established between sugars and phosphate ions [19,20,21]. The stronger H-bonded network between sugars and phosphate ions led to a significant increase of T_g_ even at high water content. In addition, the strength of the formed hydrogen bonding interactions between sugars and HPO_4_^2−^ was stronger than between sugars and H_2_PO_4_^−^ [19,20,21]. The stabilizing effect of phosphate on sugar glasses depends strongly on the pH of the phosphate buffer as for particular pH-defined proportions of NaH_2_PO_4_ and Na_2_HPO_4_ have to be mixed, resulting in different protonation states of the phosphate ions [19,21]. At a molar ratio of sugar/phosphate equal to 1, the T_g_ of Suc had been increased from 61 °C to 101 °C in the pH region 4 to 9 [19]. X-ray diffraction spectra had shown that phosphate/Suc mixtures remain amorphous up to a phosphate/Suc ratio of 0.5 [19]. In our experimental setup, we used a phosphate/Suc molar ratio of 0.33 to assure an amorphous state of the resulting mixture. The stability of sugar glasses, as determined by the increased T_g_ and the strength of the H-bonded network, can also be increased by divalent cations [58], sodium chloride [59] and sodium tetraborate [60].

The intermediate position of the melting curve of Suc/Tris between the ones of pure Suc and Suc/NaP, the value of WTC_g_ and T_g_ of dry Suc/Tris illustrated that the H-bonding interactions between Suc and Tris buffer components prevail in number and are stronger than in the pure Suc glass, but less strong than in dry Suc/NaP. The residual buffer ions, left in the Suc/buffer matrix after water removal, also changed the overall melting curve of Suc/buffer mixtures. The melting curve of pure Suc, dried from water, showed a second-order transition, while for Suc, dehydrated from Tris or NaP buffer, the transfer from a glassy to a melted state proceeded more gradually, most likely due to more gradual melting of Suc/Tris and Suc/NaP glasses with increasing temperature. Such a gradual melting could be expected if the formed H-bonded network between Suc and buffer ions was stronger and more persistent than the pure Suc glass. This curve pattern likely reflects the enthalpy relaxation of the Suc glass as the molecular mobility of Suc is increasing with an elevation in temperature [61]. In comparison to the number and strength of H-bonds (position of νOH and WTC_g_) of the common Suc dry matrix and T_g_ of the dry glass, Tris or phosphate ions increased the stability of Suc glasses in the order Suc < Suc/Tris < Suc/NaP.

The IR spectral region 1500–900 cm^−1^ is rich in vibration peaks (fingerprint region) attributed to different vibration modes of sugars arising from C–H deformation, C=O stretching, and O–H-bending vibrations. The positions of these vibration peaks are often used for evaluation of the molecular structure, vibrational properties and conformational changes of a respective sugar molecule, both in the fully hydrated and in the dry state, when involved in interactions with other molecules solvents or additives as buffer components and proteins. Even small differences in the molecular structure result in clear changes in the IR vibration peak positions [47,48].

The IR spectra of dry Suc/Tris in the region 1500–1300 cm^−1^ were very similar to that of pure Suc, dehydrated from water, indicating that interactions between Suc and Tris molecules in the dry state did not alter the vibration peak positions of Suc CH_2_ groups. In contrast, altered IR spectra of dry Suc/NaP in this region revealed that scissoring (1452 cm^−1^), rocking (1400–1300 cm^−1^) and wagging (1420 cm^−1^) vibrations of Suc CH_2_ were affected by strong H-bonding interactions between Suc and phosphate ions [48].

The IR spectral region 1300–1200 cm^−1^ is particularly sensitive to the formation of H-bonds between sugars and other molecules [47]. The peak in this region was attributed to a combination between asymmetric rocking vibration of CH_2_ and out-of-ring C–H deformation of the Suc molecule. The most prominent alteration in the Suc molecule structure in the dry state, induced by the presence of Tris and NaP components, was related to the position change of the vibration peak at 1268 cm^−1^. This peak was assigned to the rocking vibration of CH_2_ groups of Suc [48]. It can be assumed that molecules of Tris left in the Suc/buffer system after water removal were able to interact with CH_2_ groups of Suc via the formation of hydrogen bonds, thus leading to a moderate shift of the vibration peak to higher wavenumbers. The shift of this peak was much stronger for dry Suc/NaP. Consequently, H-bonding interactions between OH groups of Suc and the phosphate ion were much stronger than those between OH groups of Suc and the three OH groups of Tris, in good agreement with previous results [19,21].

In conclusion, buffer ions can stabilize Suc glasses to a high extent, and this effect might overlay the effects of LEA proteins.

### 4.2. LEA Proteins Stabilize Suc Glasses Dehydrated from Water or Tris Buffer and Influence the Molecular Structure of Dry Suc

The ability of sugars to preserve living organisms, membranes and proteins under water loss conditions is well recognized [1,2,3]. Additionally, the intracellular accumulation of highly hydrophilic LEA proteins under conditions of severe dehydration was tightly correlated with the acquisition of desiccation tolerance [24,25]. To understand the mechanisms by which LEA proteins function as cellular protectants in the dry state, it is indispensable to systematically investigate their interaction with sugars, as well as the specificity of these interactions. Published data clearly indicated that LEA proteins could stabilize membranes and other proteins during water loss, especially in the presence of sugars [24]. Only a few reports were published on the positive effect of LEA proteins on plant survival after an almost complete water loss, caused by their ability to participate in the formation of a common H-bonded network with sugars and thus increasing the T_g_ of sugar glasses [14,26,27,62]. However, this ability of LEA proteins is not unique. Similar abilities have been reported for a model glassy poly-L-lysine-Suc system [15], for a network between Suc and increasing amounts of the globular protein BSA [61] and between COR15A and sugar head groups of chloroplast galactolipids (MGDG) in the dry state [31,32].

All four LEA proteins significantly increased T_g_ of Suc to different extents, but only when dehydrated from water. The most effective protein was LEA29 at the lowest Suc/LEA protein ratio. The three closely related LEA proteins increased T_g_ of pure Suc glass in a concentration-dependent manner, with COR15A and LEA25 being more effective than LEA11. The lower efficiency of LEA11 was accompanied by an insignificant downfield shift of νOH, indicating a lower ability of LEA11 to form H-bonds with Suc OH groups than the other two proteins. A concentration-dependent stabilizing effect on Suc glass in the dry state was previously shown for the LEA protein D-7. At a Suc/protein ratio of 2, the T_g_ of Suc increased by 9 °C in the presence of D-7 compared to pure Suc, and the WTC_g_ value was decreased, resulting in stronger H-bonding interactions [14]. At the same Suc/LEA ratio, our results indicated a much stronger stabilizing effect mediated by the four investigated LEA proteins of the LEA_4 family with an increase of the T_g_ of Suc by around 30 °C and a decrease of the value of WTC_g_. It can be supposed that the interaction between Suc and LEA proteins is not strictly dependent on the molecular mass of the LEA proteins, as LEA11 and COR15A have comparable molecular masses (15.1 kDa and 9.4 kDa), while that of LEA25 is much higher (67.2 kDa) [29]. It can be concluded that the improvement of Suc glass stability is not a specific effect of LEA proteins, as a strong increase of T_g_ was also observed after the addition of globular proteins and when Suc glass was dehydrated from Tris and from NaP buffer.

The correlation analysis (Figure 8) between T_g_, WTC_g_ and νOH demonstrated that a significant correlation was found for the number of H-bonds between the OH groups of the Suc molecule and buffer ions and proteins (νOH) and the strength of these interactions (WTC_g_) that determine the molecular packing of the resulting dry Suc glass. This is an indication that both parameters, characterizing the strength of H-bonding interactions (νOH and WTC_g_), provide similar types of information about the common H-bonded network between Suc and the additives, and either one may be used for characterizing the sugar glass.

The H-bonded network strength in terms of WTC_g_ and νOH was much higher in the presence of LEA proteins than of reference proteins except for LEA29 and the WTC_g_ of COR15A in pure Suc. This is still recognizable in the presence of Tris and for LEA25 even in the presence of phosphate ions, although both buffer ions alone do already improve the network strength.

The strongest effect on the H-bonded network strength was discovered for LEA25, followed by COR15A and LEA11. It was previously shown that in the dry state, LEA25 shows less α-helicity than LEA11 and LEA11 less α-helicity than COR15A [31]. The less helical structure corresponds to larger dimensions of the protein, which might cause lower restrictions for H-bonding with Suc and more H-bond donators on the protein side not involved in intra-helix H-bonds. The authors showed that the grade of helical structure corresponded to the ability to promote H-bonded network strength with lower helicity causing a stronger network [31].

Further, the fingerprint region of Suc (1500–900 cm^−1^) was analyzed to detect LEA protein-induced alterations in the molecular structure of Suc. All LEA and globular proteins affected the position of the rocking vibration peak of CH_2_ at 1268 cm^−1^ of Suc in pure Suc glass and in both Suc/buffer combinations. For most proteins, the peak was shifted to higher wavenumbers, with the most pronounced shift caused by COR15A for pure Suc and for Suc/Tris, indicating that either the protein was able to form stronger H bonds with Suc molecules, or their number was higher.

In Suc/NaP, all LEA proteins and the two globular proteins led to an additional significant up-field shift of the peak at 1268 cm^−1^, most probably due to additional H-bonds formed between Suc and proteins. Only LEA25 caused a concentration-dependent shift of this peak to lower wavenumbers. The shift to lower wavenumbers could indicate that previously formed H bonds between Suc and phosphate ions have either been hampered or disturbed. Taken together, it can be concluded that the molecular structure of the Suc molecule in the dry state was altered by the established H-bonding interactions between Suc and proteins.

### 4.3. The Desiccation-Induced Suc Glassy State Reduced Protein Aggregation of LEA Proteins

Sugars can preserve freezing and dehydration sensitive membranes and proteins during desiccation due to their property to vitrify and to establish H-bonding interactions with membranes and proteins [3,10]. When water is removed, sugars form an amorphous glassy state that entraps membranes and proteins, restricts molecular diffusion and prevents membrane fusion and protein aggregation [9,50]. The broad and complex amide I peak of FTIR spectra is preferentially used to evaluate protein secondary structure as it represents an overlap of several vibration bands attributed to different secondary structure elements [51,56].

In the presence of osmolytes or of organic solvents, some LEA proteins acquire secondary structure, mainly α-helical components, which is also true upon dehydration [14,32]. The amide I peak of all four investigated LEA proteins, dehydrated from water, Tris or NaP buffer, was centered at 1656 cm^−1^. That indicates that in the dry state, all four LEA proteins predominantly formed α-helical structures, in line with previous reports [31]. The conformation of all dry LEA proteins was similar when entrapped in pure Suc or in Suc/buffer, but a narrower amide I contour indicated a more complex protein structure, probably due to the tighter packing of the protein. This is expected as the molecular diffusion in the amorphous glassy state is highly restricted.

Furthermore, a second peak or a well-resolved shoulder was observed at 1620 cm^−1^, characterizing the formation of intermolecular β-sheet aggregates [54,55]. Every particular LEA protein responded with a different extent of protein aggregation to complete water loss. The highest aggregation was detected for LEA11 when dehydrated from pure water and from Tris buffer, followed by LEA29, while COR15A and LEA25 displayed moderate aggregation. LEA25 was highly aggregated when dehydrated from NaP buffer alone in the absence of Suc, while the remaining proteins showed lower aggregation under this condition. Due to the highly hydrophilic character of LEA proteins, aggregation is not a common phenomenon [35]. However, aggregation of LEA proteins in the dry state has been reported for LEA17 (LEA_6 family), LEA20 and LEA35 (LEA_5 family) [50] and for LEA7 (LEA_4 family) [57]. Whereas the presence of Suc had no noticeable effect on the α-helical structure of LEA proteins, the desiccation-induced Suc glassy state significantly reduced the extent of protein aggregation due to a severe restriction of molecular diffusion [9].

## 5. Conclusions

Here we present a systematic FTIR study on the ability of four LEA proteins from the LEA_4 family in *Arabidopsis thaliana* to increase the stability of Suc glass in the dry state. To unravel the specificity of interactions, two globular proteins, BSA and LG, were included as reference proteins. In parallel, the effect of two of the most frequently used buffer components, Tris and NaP, on Suc glasses was investigated.

The strength of the Suc glass in the dry state was increased by the buffer ions in the order Suc < Suc/Tris < Suc/NaP. All investigated proteins were able to stabilize Suc glass in the dry state via establishing a common H-bonded network with Suc, visible only when Suc was dehydrated from water. The stabilizing effect of the proteins on Suc/buffer glasses was lower expressed as buffer ion effects may have overlaid protein effects. H-bonding interactions between Suc and all proteins increased in number compared to Suc alone and were stronger than intermolecular Suc-Suc H-bonding interactions. A correlation was found between the position of the stretching vibration peak of OH (νOH), representing the number of formed H-bonds and the parameter WTC_g_, characterizing the strength of these interactions, indicating that these two parameters provide similar information about the common H-bonded network. The structure of the Suc molecule in the dry state was altered by the presence of buffer ions and of all investigated proteins as evidenced by an altered contour in the spectral region (1400–1300 cm^−1^) and by the change of the rocking vibration mode position of the CH_2_ group at 1268 cm^−1^. The extent of the desiccation-induced formation of aggregates by LEA proteins was reduced by the presence of Suc.

## Data Availability

Data are available on request.

## Supplementary Materials

The following are available online at https://www.mdpi.com/article/10.3390/biom11050615/s1, Figure S1: Melting curves of dry Suc glasses obtained by plotting the position of the νOH peak against temperature. Every sample contained 10 mg/ml Suc before dehydration. Samples contained Suc and LEA11 at a ratio Suc/LEA11 = 2 and were dehydrated from H_2_O, TRIS or NaP buffer (10 mM, pH 7.4) on CaF_2_ windows. Glass transition temperatures (T_g_) were determined from the intersection of fitted regression lines in the glassy state and in the melted state.

## Abbreviations

BSA	Bovine serum albumin
FTIR	Fourier-transform infrared spectroscopy
IDP	Intrinsically disordered protein
LEA	Late embryogenesis abundant
LG	β-lactoglobulin
NaP	Sodium phosphate
Suc	Sucrose
TFE	2,2,2-trifluoroethanol
T_g_	Glass transition temperature
νOH	Vibration peak of OH groups
WTC_g_	Wavenumber–temperature coefficient in the glassy state
