# Supplementary material for: Stabilization of Dry Sucrose Glasses by Four LEA_4 Proteins from Arabidopsis thaliana
Source: Biomolecules. 2021 Apr 21;11(5):615. doi: 10.3390/biom11050615 (PMC8143093; doi:10.3390/biom11050615)
Supplement: Supplementary file 1 [file biomolecules-11-00615-s001.zip › biomolecules-1186454-supplementary.pdf]

Hincha et al. 2021

Supplemental Figure S1

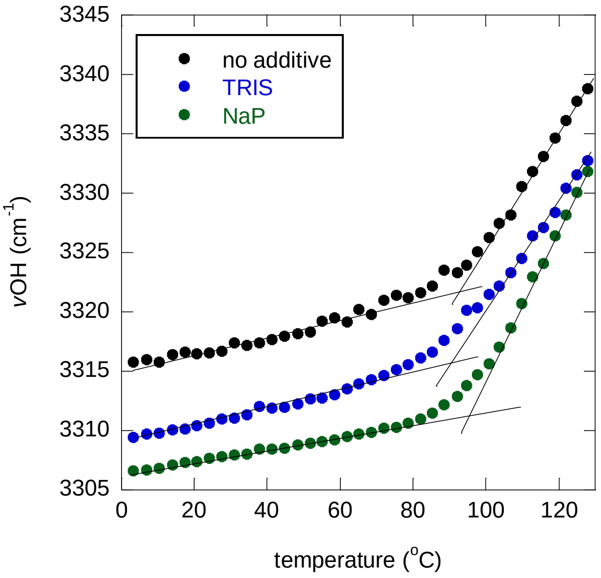

Supplemental Figure S1. Melting curves of dry Suc glasses obtained by plotting the position of the νOH peak against temperature. Every sample contained 10 mg/ml Suc before dehydration. Samples contained Suc and LEA11 at a ratio Suc/LEA11 = 2 and were dehydrated from H_2O, TRIS or NaP buffer (10 mM, pH 7.4) on CaF_2 windows. Glass transition temperatures (T_g) were determined from the intersection of fitted regression lines in the glassy state and in the melted state.
